# Comparative Transcriptome Analysis Reveals Regulatory Mechanism of Long Non-Coding RNAs during Abdominal Preadipocyte Adipogenic Differentiation in Chickens

**DOI:** 10.3390/ani12091099

**Published:** 2022-04-24

**Authors:** Weihua Tian, Xin Hao, Ruixue Nie, Yao Ling, Bo Zhang, Hao Zhang, Changxin Wu

**Affiliations:** National Engineering Laboratory for Animal Breeding, Beijing Key Laboratory for Animal Genetic Improvement, College of Animal Science and Technology, China Agricultural University, Beijing 100193, China; b20193040331@cau.edu.cn (W.T.); haoxin0331@cau.edu.cn (X.H.); s20193040548@cau.edu.cn (R.N.); lingzi@cau.edu.cn (Y.L.); chxwu@cau.edu.cn (C.W.)

**Keywords:** adipogenic differentiation, lncRNAs, competing endogenous RNA, chicken

## Abstract

**Simple Summary:**

Excessive fat deposition, particularly abdominal fat, negatively affects meat quality, feed utilization, and reproduction performance in chickens. Adipogenesis is a well-orchestrated process that involves a network of regulatory molecules. Emerging evidence has shown that long non-coding RNAs (lncRNAs) participate in mammalian adipogenesis. However, limited information is available about their role in adipogenesis in chickens. Therefore, we investigated the genome-wide identification, expression profiles, and regulatory networks of lncRNAs during adipogenic differentiation of chicken abdominal preadipocytes. We found that lncRNAs were expressed in chicken abdominal adipocytes, characterized by their differentiation stage-specific expression pattern and were involved in multiple lipid metabolism-related pathways. Several lncRNAs were identified as key lncRNAs responsible for adipogenic differentiation. These key lncRNAs could regulate lipid-related protein-encoding genes in *cis*- and *trans*-regulation manners, and/or competitively sponge miRNA to sequester it away from lipid-related genes, thereby functioning as regulators of chicken adipogenesis. These findings provide valuable information that improves our understanding of functional mechanisms of lncRNAs underlying adipogenesis and contributes to the genetic improvement of excessive abdominal fat in chickens.

**Abstract:**

Long non-coding RNAs (lncRNAs) are implicated in mammalian adipogenesis and obesity. However, their genome-wide distribution, expression profiles, and regulatory mechanisms during chicken adipogenesis remain rarely understood. In the present study, lncRNAs associated with adipogenesis were identified from chicken abdominal adipocytes at multiple differentiation stages using Ribo-Zero RNA-seq. A total of 15,179 lncRNAs were identified and characterized by stage-specific expression patterns. Of these, 840 differentially expressed lncRNAs were detected, and their *cis*- and *trans*-target genes were significantly enriched in multiple lipid-related pathways. Through weighted gene co-expression network analysis (WGCNA) and time-series expression profile clustering analysis, 14 key lncRNAs were identified as candidate regulatory lncRNAs in chicken adipogenic differentiation. The *cis*- and *trans*-regulatory interactions of key lncRNAs were constructed based on their differentially expressed *cis*- and *trans*-target genes, respectively. We also constructed a competing endogenous RNA (ceRNA) network based on the key lncRNAs, differentially expressed miRNAs, and differentially expressed mRNAs. MSTRG.25116.1 was identified as a potential regulator of chicken abdominal preadipocyte adipogenic differentiation by acting as a transcriptional *trans*-regulator of fatty acid amide hydrolase (*FAAH*) gene expression and/or a ceRNA that post-transcriptionally mediates *FAAH* gene expression by sponging gga-miR-1635.

## 1. Introduction

Chicken is widely accepted as one of the most desirable meats consumed worldwide. Abdominal fat is an important carcass composition in chickens, whereas its excessive deposition hinders profitable farming and consumer preference because of considerably reduced meat yield and quality, decreased feed efficiency, and undesirable reproductive performance [1,2,3]. Abdominal fat expands with the increased number of preadipocytes (proliferation) and the maturation of preadipocytes into adipocytes (differentiation) [4]. After birth, the development of abdominal adipose tissue occurs primarily through lipid droplet accumulation of existing adipocytes. Chicken abdominal fat deposition is particularly susceptible to genetic selection, endocrine hormones, and nutritional factors [5,6,7]. Since abdominal fat has a high heritability, genetic approaches are the most effective way to reduce carcass fat in chickens [8,9]. Therefore, it is important to study the molecular mechanisms underlying adipogenesis so that the genetic improvement regarding excessive abdominal fat can be accelerated in chickens.

To date, the genes or molecules responsible for fat deposition have been investigated using multiple genomic approaches. Long non-coding RNAs (lncRNAs) are a subclass of endogenous non-coding RNAs that are more than 200 nucleotides (nt) in length [10]. LncRNAs have been implicated in the expression regulation of protein-encoding genes at the transcriptional, post-transcriptional, and epigenetic levels that modulate a variety of diverse biological processes such as the cell cycle, cell proliferation and differentiation, lipid metabolism, tumorigenesis, and degenerative bone diseases [11,12,13,14,15]. There is also growing evidence of lncRNAs that act as important regulators during adipogenesis in mammals. For example, lncRNA SNHG1 could enhance adipogenic differentiation as well as the mRNA and protein expression levels of adipogenesis-related genes in murine bone marrow-derived mesenchymal stem cells [16]. A lncRNA existing in humans, LIPE-AS1, was antisense to lipase E, hormone sensitive type (*LIPE*) gene that encoded a hormone-sensitive lipase and shared conserved genomic organization and similar high expression in subcutaneous and visceral adipose tissue in mice. As the shortest variant of LIPE-AS1, murine Lipe antisense variant 3 (mLas-V3) was up-regulated to differentiate the bone stroma-derived pre-adipocyte cell-line model OP9 into mature adipocytes. Functionally, its knockdown blocked adipogenesis and the mRNA expression of adipogenic transcription factors [17]. Besides, there were many other lncRNAs that indeed participated in adipogenic differentiation through regulating DNA methylation and histone modifications [15]. In livestock, it was reported that lncRNAs were expressed during the development and progression of milk fat synthesis, intramuscular fat deposition, and adipogenesis. Two lncRNAs, TCONS_00082721 and TCONS_00172817, were significantly up-regulated in bovine mammary epithelial cells with high milk fat percentage compared to low milk fat percentage and were considered as potential regulators of milk fat synthesis [18]. Bovine lncFAM200B could block preadipocyte proliferation [19]. NDUFC2-AS lncRNA was highly expressed in adipose tissue and mature adipocytes in buffalo, where it facilitated adipogenic differentiation by promoting the mRNA expression level of the thyroid hormone responsive protein (*THRSP*) located upstream of NDUFC2-AS lncRNA in the genome [20]. The novel lncRNA BADLNCR1 inhibited bovine adipogenic differentiation by repressing the mRNA expression of its *cis*-target gene, glutaredoxin 5 (*GLRX5*), which was located 420 bp downstream of BADLNCR1 [21]. ADNCR, a down-regulated lncRNA during bovine adipocyte differentiation, could inhibit adipogenesis by functioning as a competing endogenous RNA (ceRNA) to sponge miR-204 and affect the expression of its target gene, sirtuin 1 (*SIRT1*), which functioned as an inhibitor of adipocyte differentiation [22]. FDNCR1 could competitively bind to miR-204 that inhibited transforming growth factor beta receptor 1 (*TGFBR1*) gene expression, thereby regulating porcine preadipocyte differentiation [23]. Finally, lncIMF2 was proven to act as a molecular sponge for miR-217 to promote the proliferation and differentiation of porcine intramuscular preadipocytes [24]. These results support the emerging importance of lncRNAs during the adipogenesis of agriculturally and economically important animals.

There are limited investigations on the roles of lncRNAs in adipogenesis in chickens. It was reported that the expressed lncRNAs were identified in abdominal fat tissues of genetically fat and lean chickens as well as differentiated and undifferentiated preadipocytes [25,26]. Moreover, lncRNA-FNIP2 could accelerate lipid synthesis through the lncRNA-FNIP2/miR-24-3p/*FNIP2* axis in chicken abdominal adipocytes [27]. Knockdown of lncAD reduced the mRNA expression of its upstream gene thioredoxin reductase 1 (*TXNRD1*) in a *cis*-regulation manner, thus leading to inhibited adipogenic differentiation and enhanced proliferation of intramuscular preadipocytes in chickens [28]. Despite these advances highlighting the relevance of lncRNAs to adipogenesis, their dynamic expression profiles and regulatory mechanisms during adipogenic differentiation have largely remained elusive. Thus, it is imperative to study the expression characteristics and regulatory roles of lncRNAs during abdominal preadipocyte differentiation in chickens.

In this study, we comprehensively analyzed the expression profiles of lncRNAs in chicken abdominal adipocytes from different differentiation stages (0, 12, 48, 72, and 120 h) using deep-sequencing. The differentially expressed lncRNAs were identified across the five developmental stages and their *cis*- and *trans*-target genes were predicted to construct *cis*- and *trans*-interaction networks. Key lncRNAs associated with adipogenic differentiation were screened via a combination of time-series expression profile clustering and weighted gene co-expression network analysis. Then, with differentially expressed miRNAs and mRNAs, the lncRNA-miRNA-mRNA competing endogenous (ceRNA) network of key lncRNAs was constructed to elucidate their regulatory mechanism underlying adipogenesis. Our study not only extends the scope of lncRNAs’ involvement in lipid metabolism, but also paves the way for lncRNA-based molecular regulation mechanisms underlying adipogenesis in chickens.

## 2. Materials and Methods

### 2.1. Ethics Statement

All experiments were approved by the Animal Welfare Committee of the State Key Laboratory for Agro-Biotechnology of the China Agricultural University (Permit Number: XK257).

### 2.2. Cell Culture and Sample Preparation

The immortalized chicken preadipocytes 2 (ICP2) cell line was obtained from the Key Laboratory of Chicken Genetics and Breeding, Ministry of Agriculture (Northeast Agricultural University). The ICP2 cells culture and adipogenic differentiation assays were conducted as previously described [29]. The cells were harvested at 0, 6, 12, 24, 48, 72, 96, and 120 h (*n* = 6) post-differentiation after washing three times in phosphate-buffered saline (PBS) (Gibco, Gaithersburg, MD, USA) and then stored at −80 °C for further RNA extraction.

### 2.3. High-Throughput RNA Sequencing (RNA-seq) Library Construction and Analysis

To investigate the dynamic expression profiles of lncRNAs and mRNAs in developing adipocytes, differentiated adipocytes at 0, 12, 48, 72, and 120 h (*n* = 3) were used for RNA-seq. The detailed procedure for cDNA library construction were described in our previous study [29]. The prepared cDNA libraries were sequenced on an Illumina Novaseq 6000 platform using the 150 bp pair-end sequencing strategy. All the generated RNA-seq data were deposited on the Sequence Read Archive (SRA) database under the accession number PRJNA732104 and were included in our published article [29].

Clean reads were yielded after removal of the adaptor, low-quality reads and reads with over 10% poly-N. The clean reads were aligned to the Galgal 6 chicken reference genome using HiSAT2 [30]. We then assembled the mapped reads into transcripts and quantified gene expression normalized by fragments per kilobase of transcript per million fragments mapped (FPKM) using the StringTie software (Johns Hopkins University, Baltimore, MD, USA) [31]. The lncRNAs were identified according to the protocols provided by Wang et al. [32]. The differentially expressed lncRNAs (DE-lncRNAs) were identified using DESeq2 [33] by a |log_2_ fold change| ≥ 1 and false discovery rate (FDR) < 0.01. The genes within 100 kb upstream and downstream of lncRNAs were considered as potential *cis*-target genes using an in-house Perl script. The potential *trans*-target genes of lncRNAs were identified based on their expression levels with an absolute Pearson correlation coefficient > 0.8 and *p* value < 0.01. The Kyoto Encyclopedia of Genes and Genomes (KEGG) pathway enrichment analysis was performed using the clusterProfiler R package (Jinan University, Guangzhou, China) [34].

### 2.4. Co-Expression Network Analysis of lncRNAs

The DE-lncRNAs expressed during chicken adipogenic differentiation were clustered using the short time-series expression miner (STEM) [35]. Expression profiles of lncRNAs were determined by their correlation coefficients. The statistical significance of the actual number of lncRNAs in each profile versus the expected number was estimated with adjusted *p* value using the Bonferroni correction.

Weighted gene co-expression network analysis (WGCNA) was employed to identify hub lncRNAs involved in adipogenesis based on the expression levels of all identified lncRNAs [36]. Briefly, a set of soft-thresholding powers (from 1 to 20) were calculated. The network was then constructed using an unsigned topological overlap matrix, a relatively large minimum module size of 30, and a merging of modules’ threshold of 0.25. Differentiation stages were used as trait files to evaluate their association with the eigengenes of each module. The modules harboring extremely significant correlations with the traits (*p* < 0.01) were used for further investigation. For these modules, we quantified gene significance (GS) and the module membership (MM), and identified hub genes with |GS| > 0.8 and |MM| > 0.8.

### 2.5. CeRNA Regulatory Network Construction

The differentially expressed miRNAs (DE-miRNAs) and differentially expressed mRNAs (DE-mRNAs) obtained from our previous study [29] were used for integrative analysis with the lncRNA data. The lncRNA-miRNA-mRNA ceRNA network was constructed based on DE-lncRNAs, DE-miRNAs, and DE-mRNAs. The microRNA response elements (MRE) of lncRNAs and mRNAs were predicted using miRanda (Memorial Sloan-Kettering Cancer Center, New York, NY, USA) [37] and TargetScan (Whitehead Institute for Biomedical Research, Cambridge, MA, USA) [38] to generate lncRNA–miRNA interaction pairs and miRNA–mRNA interaction pairs. Pearson correlation coefficient matching lncRNA–miRNA, miRNA–mRNA, and lncRNA–mRNA interaction pairs were calculated based on their expression levels with a threshold of FDR < 0.01. The ceRNA network depicting lncRNA–miRNA–mRNA was constructed using Cytoscape 3.8.2 (University of California San Diego, California, CA, USA) [39].

### 2.6. Complementary DNA (cDNA) Synthesis and Quantitative Real-Time PCR (qRT-PCR)

Eight DE-lncRNAs were selected to verify their expression in RNA-seq. For cDNA synthesis, 2 μg total RNA was reverse transcribed using the FastKing RT Kit with gDNase (TIANGEN, Beijing, China) following the manufacturer’s specifications. The qRT-PCR primers were designed using NCBI Primer-BLAST (National Center for Biotechnology Information, Bethesda, MD, USA) [40] and synthesized by SinoGenoMax (Beijing, China) (Table S1). We performed SYBR green-based qRT-PCR on a BioRad CFX96 Real-Time PCR system (BioRad, Hercules, CA, USA) in 20 μL amplification reaction volume containing 10 μL 2X Universal SYBR Green Fast qPCR Mix (ABclonal, Cambridge, MA, USA), 8.2 μL RNase-free water, 0.4 μL each of the forward and reverse primers (10 μM), and 1 μL cDNA (approximately 300 ng). The qRT-PCR protocol included an initial denaturation at 95 °C for 3 min, 40 cycles of 95 °C for 5 s, and 60 °C for 30 s. The housekeeping gene *GAPDH* served as an internal control to normalize the lncRNAs’ relative expression. The lncRNAs’ expression was calculated using the 2^−∆∆Ct^ method.

### 2.7. Statistical Analyses

All data were presented as mean ± standard deviation (SD). Statistically significant differences were determined using one-way analysis of variance (ANOVA), followed by Dunnett’s test for multiple comparisons using SPSS 23.0 software (IBM, Chicago, IL, USA). Significance was set at * *p* < 0.05 and ** *p* < 0.01. The results were illustrated using GraphPad Prism 8.3 (GraphPad Software, San Diego, CA, USA).

## 3. Results

### 3.1. Identification and Characterization of lncRNAs in Abdominal Preadipocytes

In total, 37,415 transcripts were identified in chicken abdominal adipocyte at different differentiation stages, 15,179 of which were lncRNAs. Compared to mRNAs, lncRNAs were shorter in length and characterized by a preferential length of 400 nt (Figure S1A). The open reading frame (ORF) length of the lncRNAs ranged from 40 to 160 nt, shorter than mRNAs (Figure S1B), while the majority of lncRNAs contained two exons (Figure S1C). The average expression abundance of lncRNAs was notably lower than that of mRNAs (Figure S1D). According to their genomic location, the lncRNAs were divided into four classes: 9995 intergenic lncRNAs (lincRNAs, 65.85%), 2681 intronic lncRNAs (17.66%), 2279 antisense lncRNAs (15.01%), and 224 sense lncRNAs (1.48%) that were widely distributed across chromosomes (Figure S1E,F). These results showed that lncRNAs were expressed and harbored specific genomic characteristics compared with mRNAs.

### 3.2. Differential Expression Profiles of lncRNAs during Abdominal Preadipocyte Differentiation

**Figure 1:**
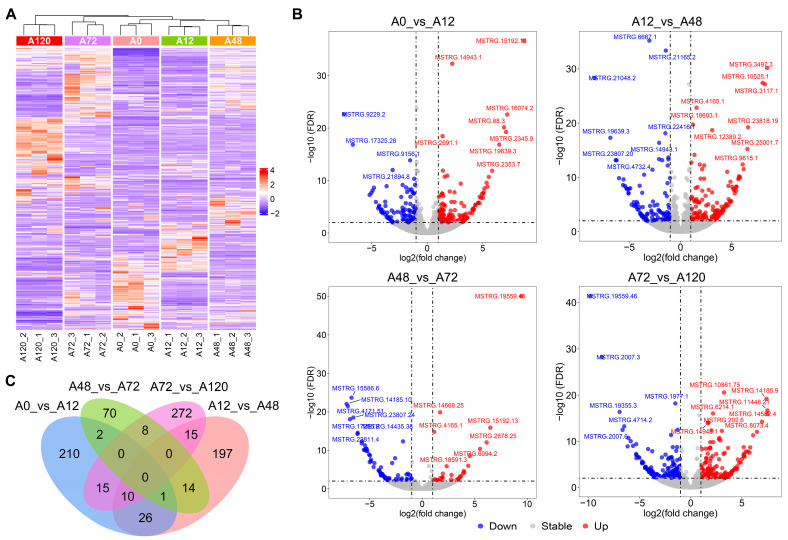
Identification of DE-lncRNAs during abdominal adipogenic differentiation in chickens. (**A**) Heatmap of DE-lncRNAs in chicken abdominal adipocytes from five differentiation stages; (**B**) volcano maps of the DE-lncRNAs in the four comparison groups (A0 vs. A12, A12 vs. A48, A48 vs. A72, and A72 vs. A120); (**C**) Venn analysis of the DE-lncRNAs from the four comparison groups.

We used pairwise comparisons (A0 vs. A12, A12 vs. A48, A48 vs. A72, and A72 vs. A120) to identify a total of 840 DE-lncRNAs during abdominal adipogenic differentiation in chickens. These included 264 DE-lncRNAs in A0 vs. A12, 263 DE-lncRNAs in A12 vs. A48, 95 DE-lncRNAs in A48 vs. A72, and 320 DE-lncRNAs in A72 vs. A120 (Figure 1A,B and Table S2). No common DE-lncRNAs were shared among the four comparison groups, while 210, 197, 70, and 272 DE-lncRNAs were specifically distributed in A0 vs. A12, A12 vs. A48, A48 vs. A72, and A72 vs. A120, respectively (Figure 1C). These results indicated that the DE-lncRNAs were characterized by differentiation stage-specific expression patterns.

### 3.3. Validation of DE-lncRNAs by qRT-PCR

**Figure 2:**
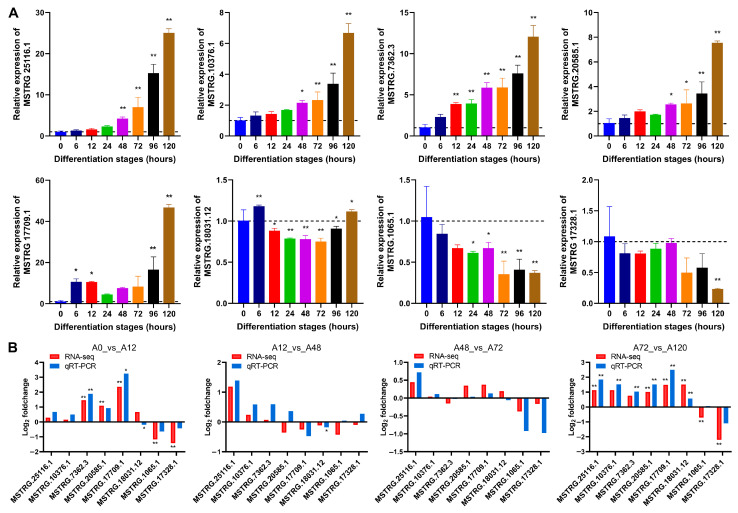
qRT-PCR validation of lncRNAs’ expression. (**A**) Detection of lncRNAs’ expression in abdominal adipocytes from eight differentiation stages using qRT-PCR. All lncRNA expression levels are shown as fold change versus that of the 0 h group; (**B**) Comparative analysis of lncRNA expression from qRT-PCR and RNA-seq data. The *x*-axis indicates the lncRNAs tested and the *y*-axis indicates the log_2_ foldchange. Red represents the expression data from RNA-seq and blue represents that from qRT-PCR. * *p* < 0.05 and ** *p* < 0.01.

To validate the expression levels of lncRNAs from RNA-seq during chicken abdominal preadipocyte differentiation, we selected eight lncRNAs to test their dynamic expression profiles by qRT-PCR. The expression of MSTRG.25116.1, MSTRG.10376.1, MSTRG.7362.3, MSTRG.20585.1, and MSTRG.17709.1 gradually increased as the chicken abdominal preadipocytes developed into adipocytes, while that of MSTRG.18031.12, MSTRG.1065.1, and MSTRG.17328.1 decreased (Figure 2A). The expression patter of these lncRNAs were consistent with RNA-seq data in the four pairwise comparison groups (Figure 2B), indicating that the RNA-seq data were reliably accurate.

### 3.4. Functional Enrichment Analysis of DE-lncRNA Cis- and Trans-Target Genes

**Figure 3:**
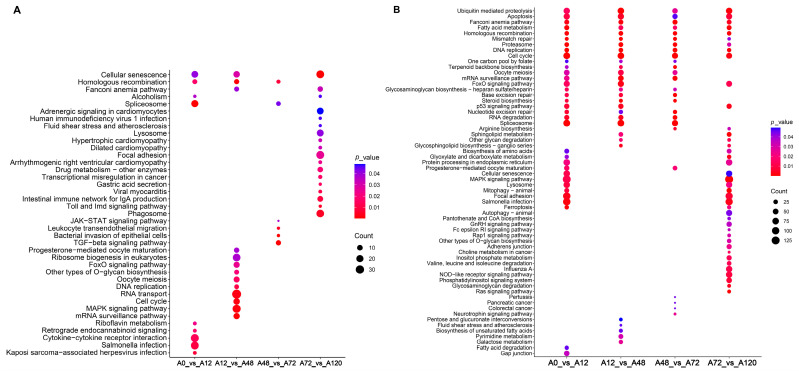
KEGG enrichment analysis of target genes of DE-lncRNAs. Significantly enriched signaling pathways of *cis*-target genes (**A**) and *trans*-target genes (**B**) of DE-lncRNAs in the four comparison groups.

To explore the potential regulatory roles of DE-lncRNAs, their *cis*- and *trans*-target genes were predicted, and functional enrichment analysis was performed. Our results suggested that the *cis*-target genes of these DE-lncRNA were significantly enriched in lipid-related signaling pathways, including the MAPK and FoxO signaling pathways in A12 vs. A48, and TGF-β and JAK-STAT signaling pathways in A48 vs. A72 (Figure 3A and Tables S3 and S4). The *trans*-target genes of DE-lncRNAs in A0 vs. A12 were significantly enriched in several lipid-related signaling pathways including fatty acid metabolism, MAPK signaling pathway, FoxO signaling pathway, and fatty acid degradation. The significantly enriched lipid-related signaling pathways of the *trans*-target genes of DE-lncRNAs in A12 vs. A48 included steroid biosynthesis, FoxO signaling pathway, fatty acid metabolism, and unsaturated fatty acid biosynthesis. The *trans*-target genes of DE-lncRNAs in A48 vs. A72 were steroid biosynthesis and fatty acid metabolism, while those of DE-lncRNAs in A72 vs. A120 were enriched in MAPK signaling pathway, sphingolipid metabolism, fatty acid metabolism, NOD-like receptor signaling pathway, and FoxO signaling pathway. Of these significantly enriched pathways, fatty acid metabolism was common in all comparison groups and predicted to drive the entire process of adipogenic differentiation in chickens. Only fatty acid degradation was present in A0 vs. A12, biosynthesis of unsaturated fatty acids was specifically found in A48 vs. A72, and the NOD-like receptor signaling pathway was unique to A72 vs. A120 (Figure 3B, Tables S3 and S4). Together, these results suggested the regulatory roles of lncRNAs in the adipogenic differentiation of chicken abdominal preadipocyte.

### 3.5. Short Time-Series Expression Analysis of DE-lncRNAs

**Figure 4:**
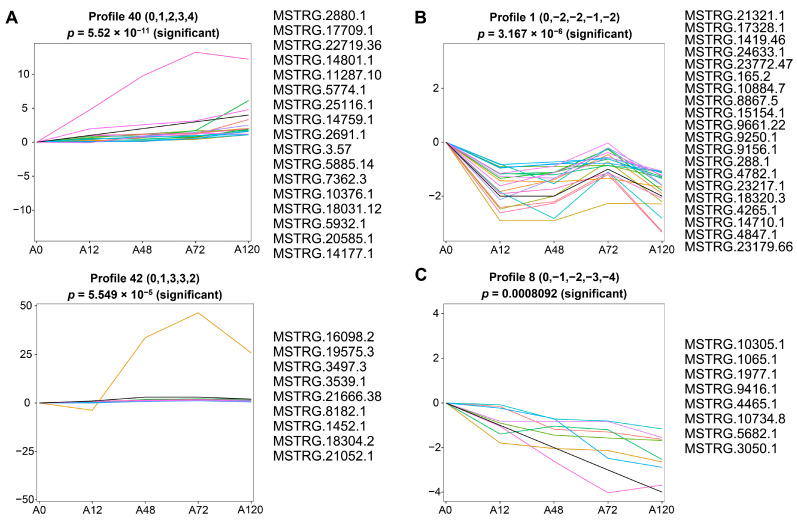
Short time-series expression analysis of DE-lncRNAs during abdominal adipogenic differentiation. (**A**) The expression profiles of DE-lncRNAs in cluster 1 containing profile 40 and profile 42; (**B**) the expression profiles of DE-lncRNAs in cluster 2 containing profile 1; (**C**) the expression profiles of DE-lncRNAs in cluster 3 containing profile 8. The *x*-axis indicates differentiation stages including 0 h (A0), 12 h (A12), 48 h (A48), 72 h (A72), and 120 h (A120). The *y*-axis indicates the log_2_ foldchange in their expression. The colored lines represent the expression pattern of lncRNAs.

To identify the co-expressed lncRNAs associated with adipogenesis in chickens, we analyzed short time-series expression data of DE-lncRNAs using a short time-series expression miner (STEM). The results suggested that these DE-lncRNAs were partitioned into three clusters derived from the red, green, and blue profiles, of which four profiles (40, 42, 1, and 8) had a statistically significant number of lncRNAs assigned (Figure S2 and Table S5). The expression pattern of DE-lncRNAs in cluster 1 containing profiles 40 and 42 was overall up-regulated during abdominal adipogenic differentiation in chickens. In particular, profile 40 contained 17 DE-lncRNAs that exhibited a sequentially increased expression pattern. The expression of profile 42, which contained nine DE-lncRNAs, displayed gradual up-regulation until 48 h, followed by a stabile expression pattern at 72 h post-differentiation, with a slight reduction in expression at 120 h post-differentiation that was still higher than at 0 and 12 h post-differentiation (Figure 4A). The expression of cluster 2, which included profile 1 that contained 20 DE-lncRNAs, decreased until 48 h post differentiation, increased at 72 h post differentiation, but was still lower than preadipocytes, and then decreased at 120 h post differentiation to the expression level at 48 h post-differentiation (Figure 4B). Cluster 3 included profile 8, which was composed of eight DE-lncRNAs and demonstrated continuously decreased expression throughout abdominal adipogenic differentiation (Figure 4C).

### 3.6. Weighted Gene Co-Expression Network Analysis (WGCNA) of All Expressed lncRNAs

**Figure 5:**
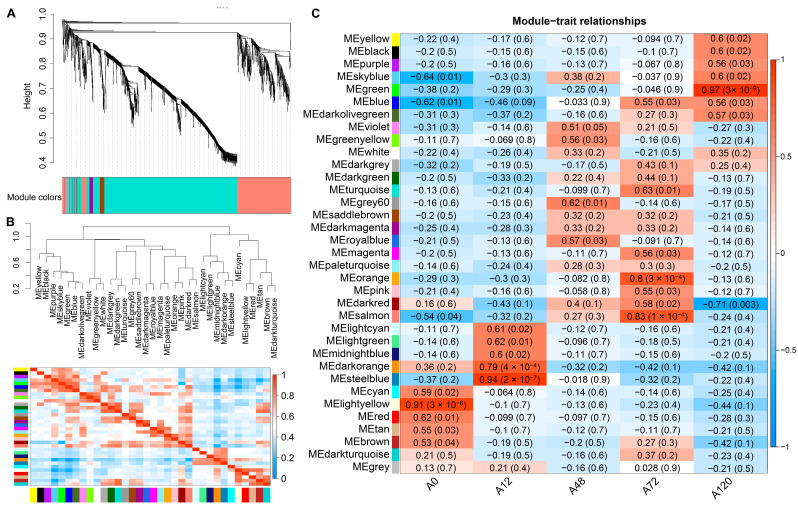
WGCNA of all expressed lncRNAs in chicken abdominal preadipocytes and differentiated adipocytes. (**A**) Hierarchical clustering dendrograms showing 35 modules of co-expressed lncRNAs. Color bar indicates the modules of co-expressed lncRNAs; (**B**) visualization of the eigengene network representing the relationships among the modules; (**C**) module-trait association heatmap where each row corresponds to a module eigengene and the columns correspond to a trait. Each cell contains the correlation and *p* value for the corresponding differentiation stages. The scale bar indicates the color coding for the correlations, with blue to red indicating low to high correlations, respectively.

To further identify the co-expressed hub lncRNAs responsible for chicken abdominal preadipocyte differentiation, a total of 15,179 lncRNAs were subjected to WGCNA based on their expression levels in chicken abdominal preadipocytes from five differentiation stages. We chose a soft-thresholding power value of 5 to analyze the network topology because it is the lowest power at which the scale-free topology fit index curve flattens out upon reaching an R^2^ cut-off of 0.85 (Figure S3). Ultimately, 35 modules were identified, ranging in size from 39 lncRNAs in the dark magenta module to 1494 lncRNAs in the turquoise module (Figure 5A,B). Seven of these modules had significant module-trait associations (*p* < 0.01), including the light-yellow module, which was significantly positively associated with 0 h post differentiation (preadipocytes) (*p* = 3 × 10^−6^); the dark-orange (*p* = 4 × 10^−4^) and steel-blue modules (*p* = 2 × 10^−7^), which were significantly positively associated with 12 h post differentiation; and the orange (*p* = 3 × 10^−4^) and salmon modules (*p* = 1 × 10^−4^), which were significantly positively associated with 72 h post differentiation; as well as the dark-red module (*p* = 0.003), which was significantly negatively correlated with 120 h post differentiation and the green module (*p* = 3 × 10^−9^), which was significantly positively correlated with 120 h post differentiation (Figure 5C). These results suggested that the expression profiles of lncRNAs in the seven modules might respond to the adipogenic differentiation of chicken abdominal preadipocytes.

### 3.7. Identification of Hub lncRNAs

**Figure 6:**
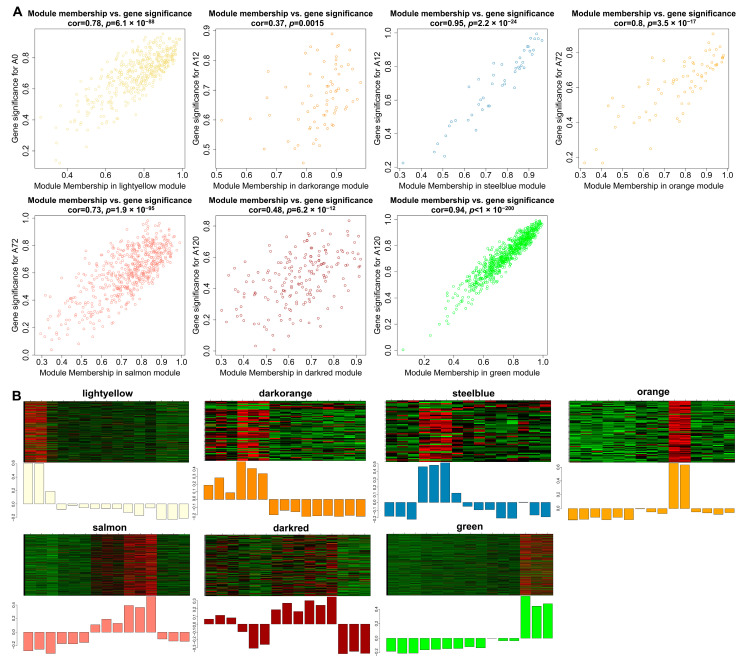
Visualization of GS vs. MM and lncRNAs’ expression levels in seven modules. (**A**) A scatterplot of GS for differentiation stage vs. MM in seven modules. The cor means the correlation between GS and MM. The *p* value represents the significance of correlation between GS and MM; (**B**) Heatmap and bar-plot representing the expression levels of lncRNAs in 15 samples (from left to right: A0-1, A0-2, A0-3, A12-1, A12-2, A12-3, A48-1, A48-2, A48-3, A72-1, A72-2, A72-3, A120-1, A120-2, and A120-3) in seven modules. The color ranging from green to red in the heatmap indicates low to high expression levels.

Gene significance (GS) represented the correlation between genes and traits, and the higher absolute value of GS indicates that the gene is more significant regarding the biological question of interest. The module membership (MM) represented the correlation of the module eigengene with the gene expression profile, and the higher absolute value of MM indicates that the gene has a more relevant relationship with the module eigengene. Using the GS and MM measurements, we then identified hub lncRNAs that had a high correlation with the abdominal adipogenic differentiation stages (|GS| > 0.8), as well as the high correlation of the module eigengene and the lncRNAs’ expression profile (|MM| > 0.8) in the above seven modules, significantly linking to differentiation stages. For example, in the light-yellow module, GS and MM were significantly highly correlated (cor = 0.78, *p* = 6.1 × 10^−88^), illustrating that the lncRNAs in the light-yellow module were significantly positively associated with 0 h post differentiation (preadipocytes) (Figure 6A). Accordingly, the expression levels of these lncRNAs achieved an uppermost peak in preadipocytes, consistent with the significantly positive correlation of the light-yellow module with preadipocytes (correlation = 0.91, *p* = 3 × 10^−6^; Figure 6B). A total of 463 hub lncRNAs were identified in the seven modules, among which, 158 hub lncRNAs were differentially expressed (Table S6). Of these lncRNAs, 14 differentially expressed hub lncRNAs were also validated using short time-series expression analysis. Of which, eight lncRNAs from the green module were found in profile 40 as well, while there were also four lncRNAs, one lncRNA, and one lncRNA from the light-yellow module in profile 1, profile 8, and profile 40, respectively (Table S6). Therefore, the 14 differentially expressed hub lncRNAs were considered as key lncRNAs for further investigation during adipogenic differentiation in chickens.

### 3.8. Cis- and Trans-Regulatory Networks of Key lncRNAs

To further explore the molecular regulatory mechanism of the identified key lncRNAs during adipogenesis, we first constructed *cis*- and *trans*-regulatory networks based on the key lncRNAs and their differentially expressed *cis*- and *trans*-target genes. A total of 12 *cis*-regulatory interaction pairs comprising nine key lncRNAs and 12 differentially expressed *cis*-target genes were detected (Figure S4A and Table S7). There were 2020 *trans*-regulatory interaction pairs containing 14 key lncRNAs and 410 differentially expressed *trans*-target genes. Interestingly, 95 of these pairs included 10 key lncRNAs and 15 differentially expressed lipid-related *trans*-target genes (Figure S4B and Table S7). Of the 15 *trans*-target genes, ATP-binding cassette, sub-family A (ABC1), member 1 *(ABCA1*), acyl-CoA synthetase short-chain family member 2 (*ACSS2*), 1-acylglycerol-3-phosphate O-acyltransferase 2 (*AGPAT2*), cytochrome P450 family 26 subfamily B member 1 (*CYP26B1*), DDHD domain containing 1 (*DDHD1*), ENSGALG00000020485, *FAAH*, fatty acid binding protein 4 (*FABP4*), and methylcrotonoyl-CoA carboxylase 1 (*MCCC1*) were involved in *trans*-regulatory interactions with each of MSTRG.17709.1, MSTRG.14759.1, MSTRG.18031.12, MSTRG.2880.1, MSTRG.20585.1, MSTRG.25116.1, MSTRG.11287.10, and MSTRG.10376.1. These results indicated that lncRNAs might likely serve as a *trans*-regulator to function in adipogenic differentiation of chicken abdominal preadipocytes.

### 3.9. CeRNA Network Construction of Key lncRNAs

**Figure 7:**
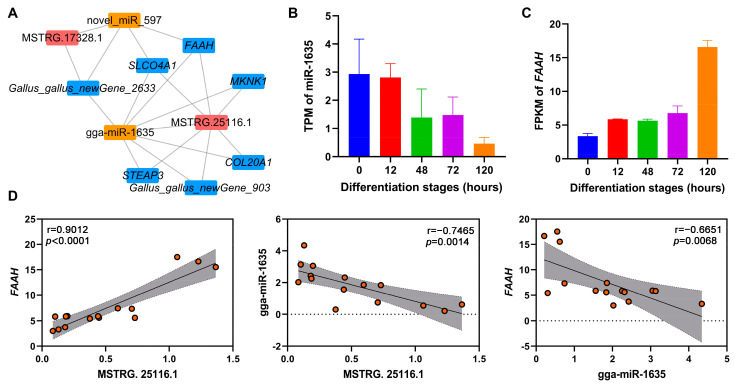
The lncRNA–miRNA–mRNA ceRNA networks mediated by key lncRNAs. (**A**) The ceRNA networks of key lncRNAs, DE-miRNAs, and DE-mRNAs; (**B**,**C**) RNA-seq expression analysis of gga-miR-1635 and *FAAH* gene in chicken abdominal preadipocytes from different differentiation stages; (**D**) correlation analysis of the expression levels of MSTRG.25116.1, gga-miR-1635, and *FAAH* gene from RNA-seq during adipogenic differentiation in chicken abdominal preadipocytes. The red dots indicate the expression levels of genes that were represented on *y*-axis.

To identify potential ceRNA networks during adipogenic differentiation of chicken abdominal preadipocytes, we constructed ceRNA networks of DE-lncRNAs, DE-miRNAs, and DE-mRNAs in four pairwise comparisons (Figure S5 and Table S8). Two of the key lncRNAs (MSTRG.25116.1 and MSTRG.17328.1) were found in the ceRNA regulatory networks. MSTRG.25116.1 was found to sponge gga-miR-1635, which could mediate the post-transcriptionally regulation of its potential target lipid-related gene, fatty acid amide hydrolase (*FAAH*; Figure 7A). Based on the RNA-seq data, gga-miR-1635 showed decreased expression as chicken abdominal preadipocytes differentiated, while *FAAH* expression showed an overall increase (Figure 7B,C). Moreover, the expression levels of MSTRG.25116.1 and *FAAH* were significantly positively correlated (r = 0.9012, *p* < 0.0001), while that of MSTRG.25116.1 and gga-miR-1635 (r = −0.7465, *p* = 0.0014) and gga-miR-1635 and *FAAH* (r = −0.6651, *p* = 0.0068) were both significantly negatively correlated (Figure 7D). Together, these results suggested that MSTRG.25116.1 could function as a ceRNA to increase *FAAH* mRNA expression by sponge gga-miR-1635 and thus regulate adipogenic differentiation of chicken abdominal preadipocytes.

## 4. Discussion

In recent decades, excessive deposition of body fat, particularly abdominal fat, has been accompanied by intensive genetic selection for rapid growth rate and high meat yield in broiler chickens [41]. Excessive fat is often discarded as waste and presents an obstacle to profitable farming practices. It is well-acknowledged that adipogenesis is a well-orchestrated process that involves a cascade of regulatory molecules, such as noncoding RNAs that represent the vast majority of the total genome. As a type of noncoding RNA, lncRNAs play profound regulatory and functional roles in mammalian lipid metabolism, thereby gaining prominence as a key adipogenic driver [14,42,43]. However, the physiological role of lncRNAs in avian adipogenesis has remained largely uncharacterized. In the present study, we compared the differences in lncRNA expression during chicken abdominal adipocyte differentiation to identify key lncRNAs associated with adipogenesis and their potential regulatory mechanisms.

Our findings showed that lncRNAs identified in chicken abdominal adipocytes shared similar genomic characteristics to those in mammals, including relatively short total lengths and ORF sequences, fewer exons, and lower expression levels than protein-encoding genes [44,45,46]. Most of the differentially expressed lncRNAs were characterized by their differentiation stage-specific expression, which suggested the complexity of regulatory mechanisms mediated by lncRNAs during adipogenesis in chickens. It is well established that lncRNA could mediate both the transcriptional regulation of neighboring protein-encoding genes on the same chromosome from which they are transcribed (*cis*-acting), as well as influence protein-encoding genes on other chromosomes (*trans*-acting) [47]. Here, functional enrichment analysis of *cis*-target genes of differentially expressed lncRNAs revealed significant enrichment of the signaling pathways related to lipid metabolism in A12 vs. A48 and A48 vs. A72 comparison groups, indicating that lncRNAs might *cis*-regulate adipogenic differentiation from 12 to 72 h post differentiation. Interestingly, *trans*-target genes were significantly enriched in multiple signaling pathways involving lipid metabolism in all comparison groups, wherein a common signaling pathway was fatty acid metabolism, which is a well-acknowledged crucial player in fatty acid biosynthesis and elongation, β-oxidation, and acyl-CoA synthesis. These results suggested that lncRNAs could govern the overall period of chicken adipogenic differentiation most likely by *trans*-regulating protein-encoding gene expression.

Generally, a discrete biological process depends upon complex interactions that involve a network of functional genes. Co-expression network analysis allows us to simultaneously identify, cluster, and explore thousands of genes that shared similar expression patterns across multiple conditions. This helps to identify co-expressed genes that can form clusters or modules though their shared function or involvement in the same regulatory pathway [48]. Based on the lncRNA expression data, we identified significant temporal expression profiles of lncRNAs using STEM, a tool specialized for short time-series expression analysis [35]. Three clusters containing 54 lncRNAs associated with chicken abdominal preadipocyte differentiation were enriched. The lncRNAs in cluster 1 showed an overall upregulation as chicken abdominal preadipocyte differentiation progressed, suggesting a sustained positive effect on adipogenic differentiation. LncRNA expression in cluster 2 exhibited an overall decrease when compared to preadipocyte, except for a significant increase at 72 h post-differentiation, suggesting that these lncRNAs dynamically regulate adipogenic differentiation in chickens. The lncRNAs in cluster 3 were gradually downregulated as adipogenic differentiation progressed, suggesting they have an inhibiting effect over the entire differentiation process.

The significant modules and hub lncRNAs related to adipogenic differentiation in chickens were identified using WGCNA, which is specialized to find clusters (modules) of highly correlated genes and relate the modules to external sample traits [36]. In our study, seven modules were significantly associated with the differentiation stages of chicken abdominal preadipocytes, in which 158 lncRNAs were identified as hub lncRNAs. These results suggest that these lncRNAs might be master regulators of chicken adipogenesis. Combined with the clustered lncRNAs from short time-series expression analysis and hub lncRNAs generated by WGCNA, we identified a subset of key lncRNAs involving chicken abdominal preadipocyte differentiation and revealed their potential *cis*- and *trans*-regulatory roles. Among *cis*-acting genes of key lncRNAs, the lamin B1 (*LMNB1*) gene could encode the lamin B1 protein, which is a constituent of nuclear lamina and has been proved to play an essential role in maintaining nuclear architecture by regulating gene expression and modulating chromatin positioning [49,50]. The *LMNB1* gene has been linked to lipid metabolism in transgenic mice overexpressing *LMNB1* that showed a reduced expression of lipid synthesis genes and myelin-enriched lipid levels in an age-dependent manner [51]. However, there is no current evidence on whether other *cis*-target genes of these key lncRNAs are related to lipid metabolism. Interestingly, several *trans*-target genes of key lncRNAs were involved in lipid metabolism, such as *FABP4*, *ABCA1*, and StAR-related lipid transfer domain-containing 5 (*STARD5*), which encode key enzymes for lipid uptake and transport; acyl-CoA synthetase long-chain family member 1 (*ACSL1*), *ACSS2*, ELOVL fatty acid elongase 7 (*ELOVL7*), *AGPAT2*, and *MCCC1* that are responsible for de novo lipogenesis; carnitine palmitoyltransferase 1A (*CPT1A*), which encodes a rate-limiting enzyme in mitochondrial fatty acid oxidation; *DDHD1*, DDHD domain containing 2 (*DDHD2*), and *FAAH*, which govern fatty acid hydrolysis; and *CYP26B1*, which is a master mediator of cholesterol and steroids synthesis. Collectively, we hypothesized that these key lncRNAs do participate in chicken adipogenic differentiation by *trans*-regulating the transcriptional activity of protein-encoding genes associated with lipid metabolism.

Growing evidence supports the ceRNA hypothesis that lncRNAs can post-transcriptionally regulate the expression of protein-encoding genes by acting as decoys that competitively adsorb miRNAs to sequester miRNAs away from their target protein-encoding genes [13]. As a class of endogenous non-coding RNAs, miRNA could regulate gene expression by complementarily binding to miRNA response elements of target genes at a post-transcriptional level and thus play important regulatory roles in adipocyte differentiation [52]. To comprehensively probe how the ceRNA regulatory interactions of key lncRNAs affect adipocyte differentiation in chickens, we successfully constructed a ceRNA network of key lncRNAs, differentially expressed miRNAs, and mRNAs. The lncRNA MSTRG.25116.1 exhibited a gradual increase in expression that followed the stages of adipogenic differentiation in chicken abdominal preadipocytes, suggesting it might play a crucial role during adipogenesis. MSTRG.25116.1 could sponge gga-miR-1635, which potentially targets *FAAH*. *FAAH* encodes one of the best-characterized enzymes involved in the hydrolysis of bioactive lipids and serves as a potential therapeutic target for the treatment of obesity [53]. The genetic variation in *FAAH* expression is associated with the hypophagic effects of leptin and obesity [54,55,56]. It has been demonstrated that *FAAH* was up-regulated in the abdominal fat of genetically fat chickens when compared with lean chickens [26]. Consistent with this report, our study revealed that *FAAH* was overall up-regulated in chicken abdominal adipocytes versus preadipocytes, indicating its important function during chicken adipogenesis. Taken together, MSTRG.25116.1 could serve as a molecular sponge that competitively binds to gga-miR-1635 to promote the post-transcriptional expression of *FAAH*, ultimately functioning as a positive regulator of adipogenic differentiation in chickens. Interestingly, MSTRG.25116.1 was also found to directly regulate the transcriptional activity of *FAAH* in a *trans*-regulation manner in our constructed regulatory network analysis. Further validation of the MSTRG.25116.1/gga-miR-1635/*FAAH* axis and/or MSTRG.25116.1/*FAAH* axis during chicken adipogenesis is needed to expand our understanding of this transcriptional and post-transcriptional regulatory networks.

## 5. Conclusions

We reported the genome-wide identification, dynamic expression profiles, and regulatory mechanisms of lncRNAs in chicken abdominal adipocytes throughout their differentiation. A total of 840 differentially expressed lncRNAs were identified and involved in multiple signaling pathways related to lipid metabolism through *cis*- and *trans*-regulatory interactions. Cooperated time series expression profile clustering analysis with co-expression network analysis identified 14 key lncRNAs as potential regulators of adipogenesis in chickens. Of these, MSTRG.25116.1 was gradually up-regulated during chicken abdominal preadipocytes’ adipogenic differentiation and could participate in adipogenic differentiation as a *trans*-acting regulator of the transcriptional activity of the fatty acid amide hydrolase (*FAAH*) gene, and/or a ceRNA to competitively sponge gga-miR-1635 to post-transcriptionally increase *FAAH* expression in chickens. These results provide new insights and valuable resources for further research of the molecular mechanisms underlying avian adipogenesis.

## Data Availability

All RNA-seq data supporting the results of this article have been deposited in the National Center for Biotechnology Information (NCBI) Sequence Read Archive (SRA) database under accession number PRJNA732104.

## Supplementary Materials

The following supporting information can be downloaded at: https://www.mdpi.com/article/10.3390/ani12091099/s1. Figure S1: Characteristics of lncRNAs expressed in chicken abdominal adipocytes during adipogenic differentiation; Figure S2: Profiles were constructed using STEM analysis of all DE-lncRNAs during abdominal adipogenic differentiation; Figure S3: Network topology analysis for soft-thresholding powers from 1 to 20; Figure S4: The *cis*- and *trans*-regulatory networks of key lncRNAs and DE-mRNAs; Figure S5: The lncRNA-miRNA-mRNA ceRNA networks in chicken abdominal adipocytes from different differentiation stages; Table S1: Primers used in this study; Table S2: Differential expressed lncRNAs in chicken abdominal adipocytes during adipogenesis; Table S3: Prediction of *cis*- and *trans*-target genes of differentially expressed lncRNAs; Table S4: KEGG signaling pathway enrichment analysis of differentially expressed lncRNAs during adipogenesis; Table S5: Short time-series expression analysis of differentially expressed lncRNAs; Table S6: Weighted gene co-expression network analysis of all expressed lncRNAs; Table S7: Regulatory interaction of key lncRNAs and their differentially expressed target genes; Table S8: Competing endogenous RNA regulatory network of key lncRNAs.
