# Supplementary material for: Comparative Transcriptome Analysis Reveals Regulatory Mechanism of Long Non-Coding RNAs during Abdominal Preadipocyte Adipogenic Differentiation in Chickens
Source: Animals (Basel). 2022 Apr 24;12(9):1099. doi: 10.3390/ani12091099 (PMC9101879; doi:10.3390/ani12091099)
Supplement: Supplementary file 1 [file animals-12-01099-s001.zip › Supplementary Figures.pdf]

Supplementary Figures

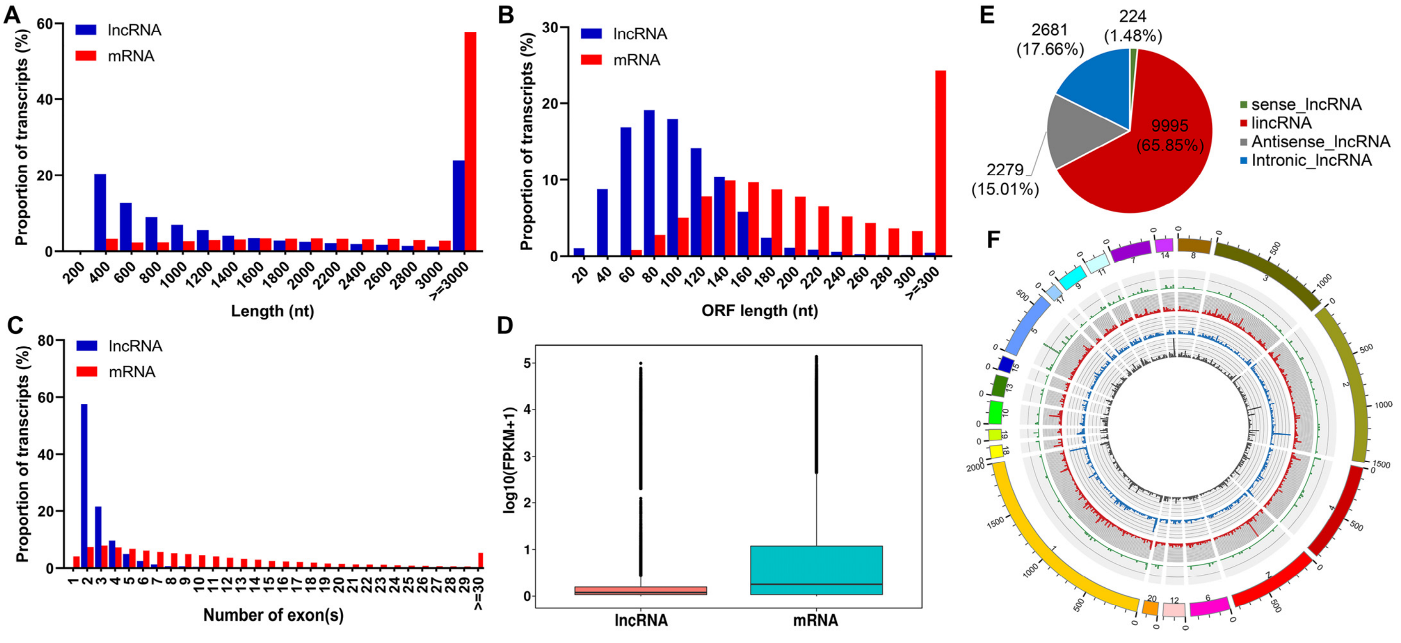

Figure S1. Characteristics of lncRNAs expressed in chicken abdominal adipocytes during adipogenic differentiation. (**A**) Length distribution of lncRNAs and mRNAs. (**B**) ORF length distribution of lncRNAs and mRNAs. (**C**) LncRNAs and mRNAs exon numbers. (**D**) Transcript FPKM distribution of lncRNAs and mRNAs. (**E**) Classification of lncRNAs expressed in chicken abdominal adipocytes. (**F**) A circos plot showing the distributions of different types of lncRNAs on chicken chromosomes.

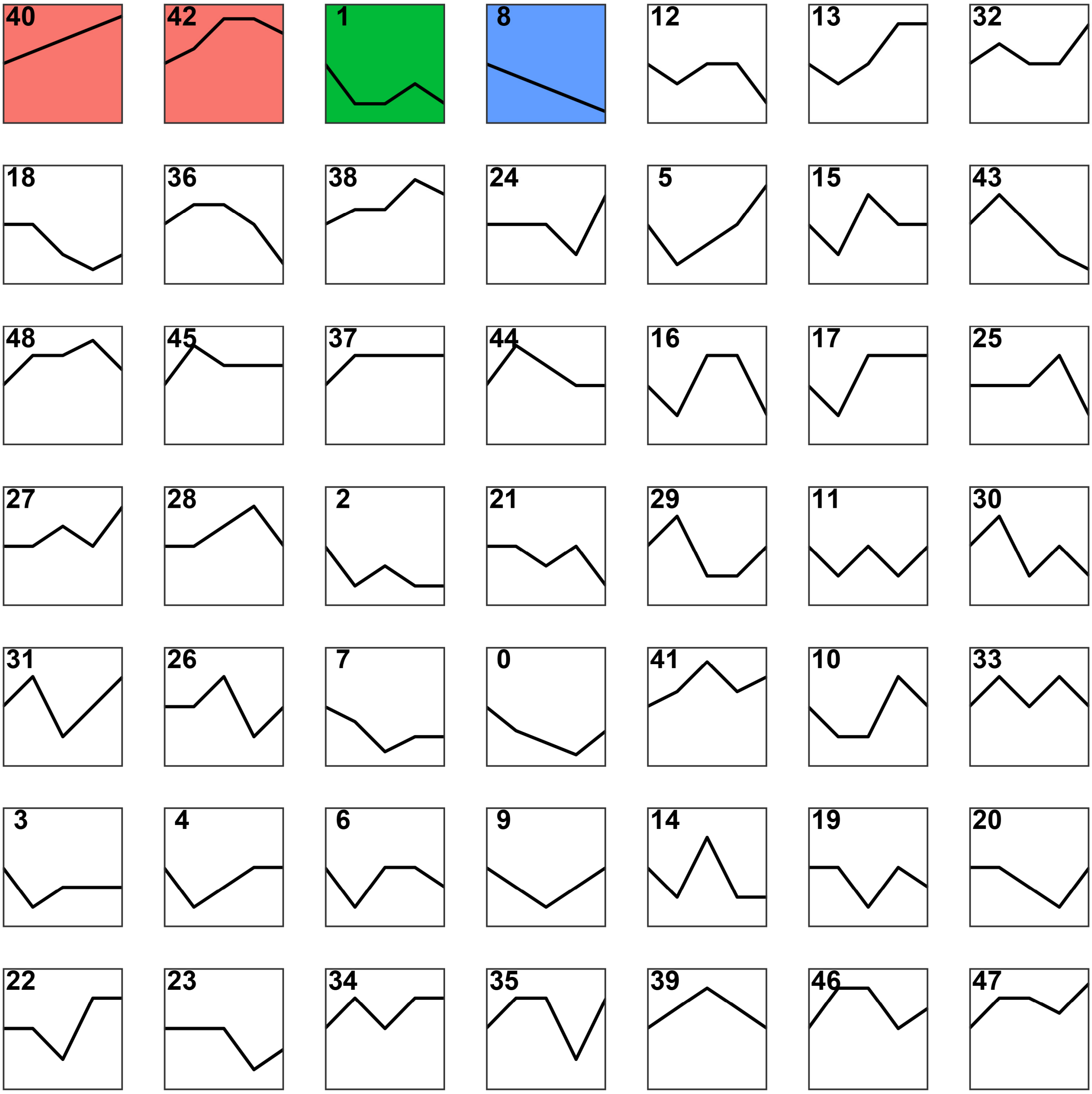

Figure S2. Profiles were constructed using STEM analysis of all DE-lncRNAs during abdominal adipogenic differentiation. The numbers in the top left corner of the profile boxes are the profile ID number. The profiles with the same color represent a single cluster.

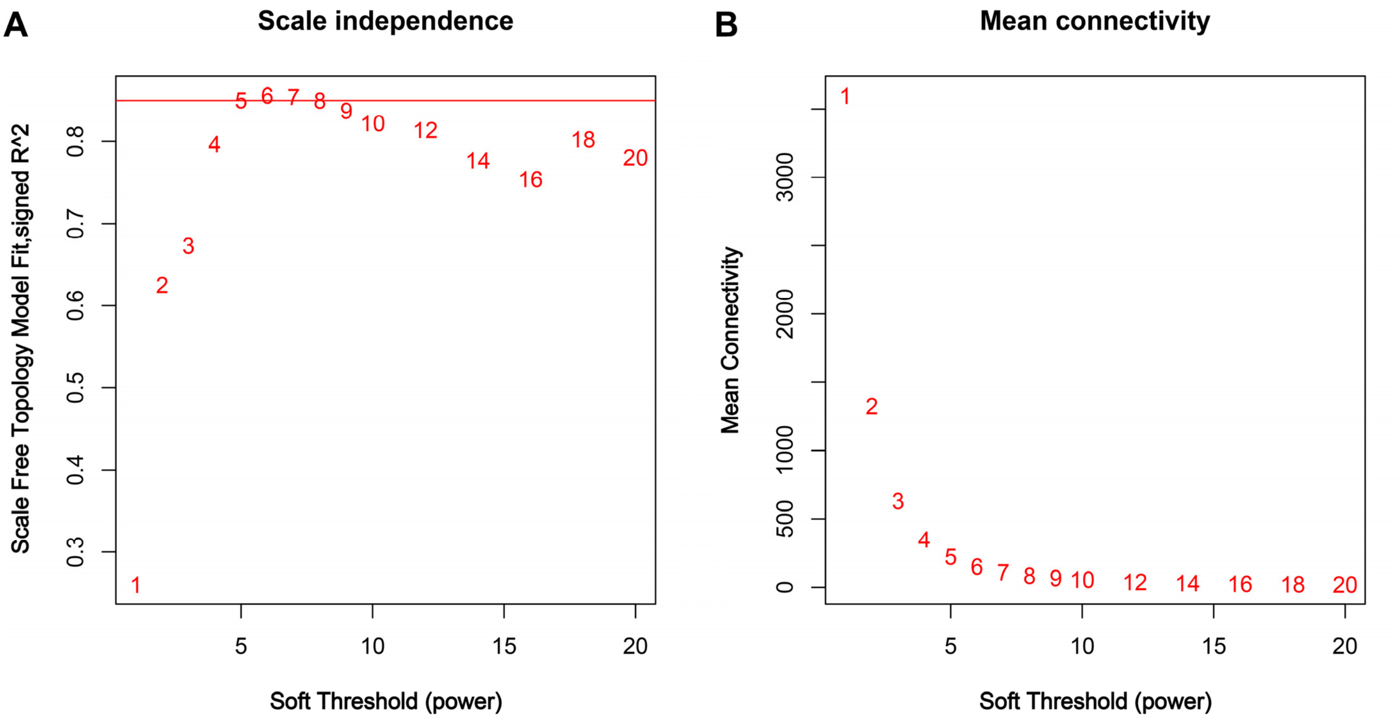

Figure S3. Network topology analysis for soft-thresholding powers from 1 to 20. (**A**) Effects of the soft-thresholding power (x-axis) on the scale-free fit index (y-axis). (**B**) Effects of the soft-thresholding power (x-axis) on the mean connectivity (degree, y-axis).

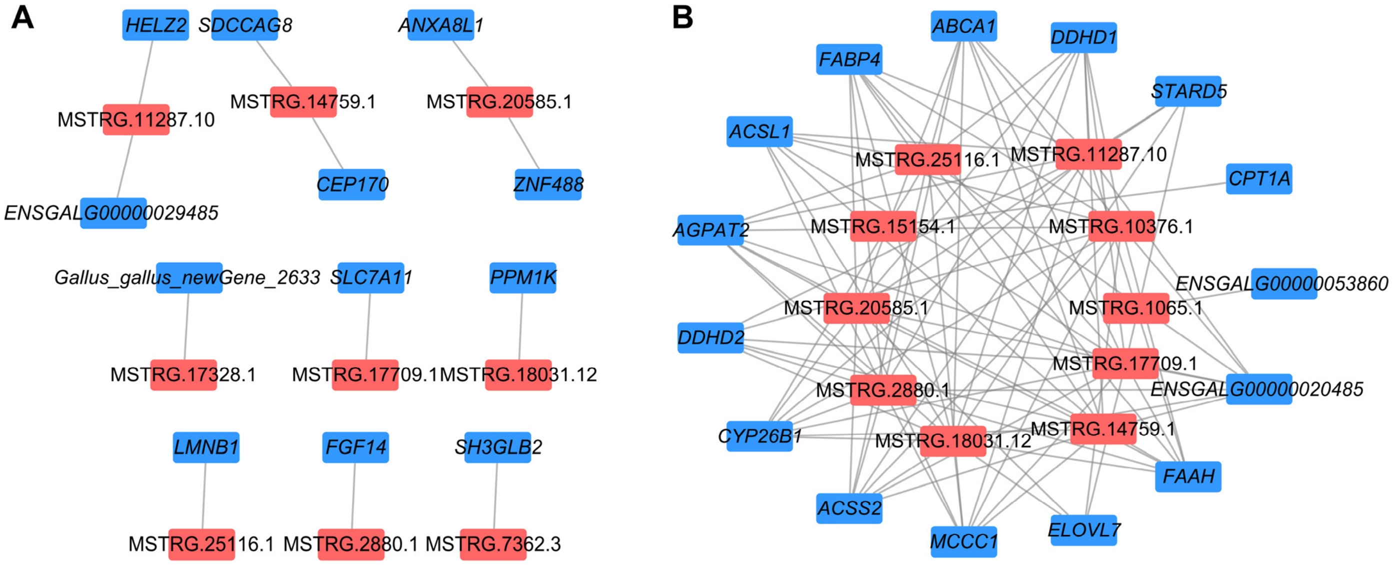

Figure S4. The *cis*- and *trans*-regulatory networks of key lncRNAs and DE-mRNAs. (**A**) The *cis*-regulatory networks of key lncRNAs and their differentially expressed *cis*-target genes. (**B**) The *trans*-regulatory networks of key lncRNAs and their differentially expressed *trans*-target genes.

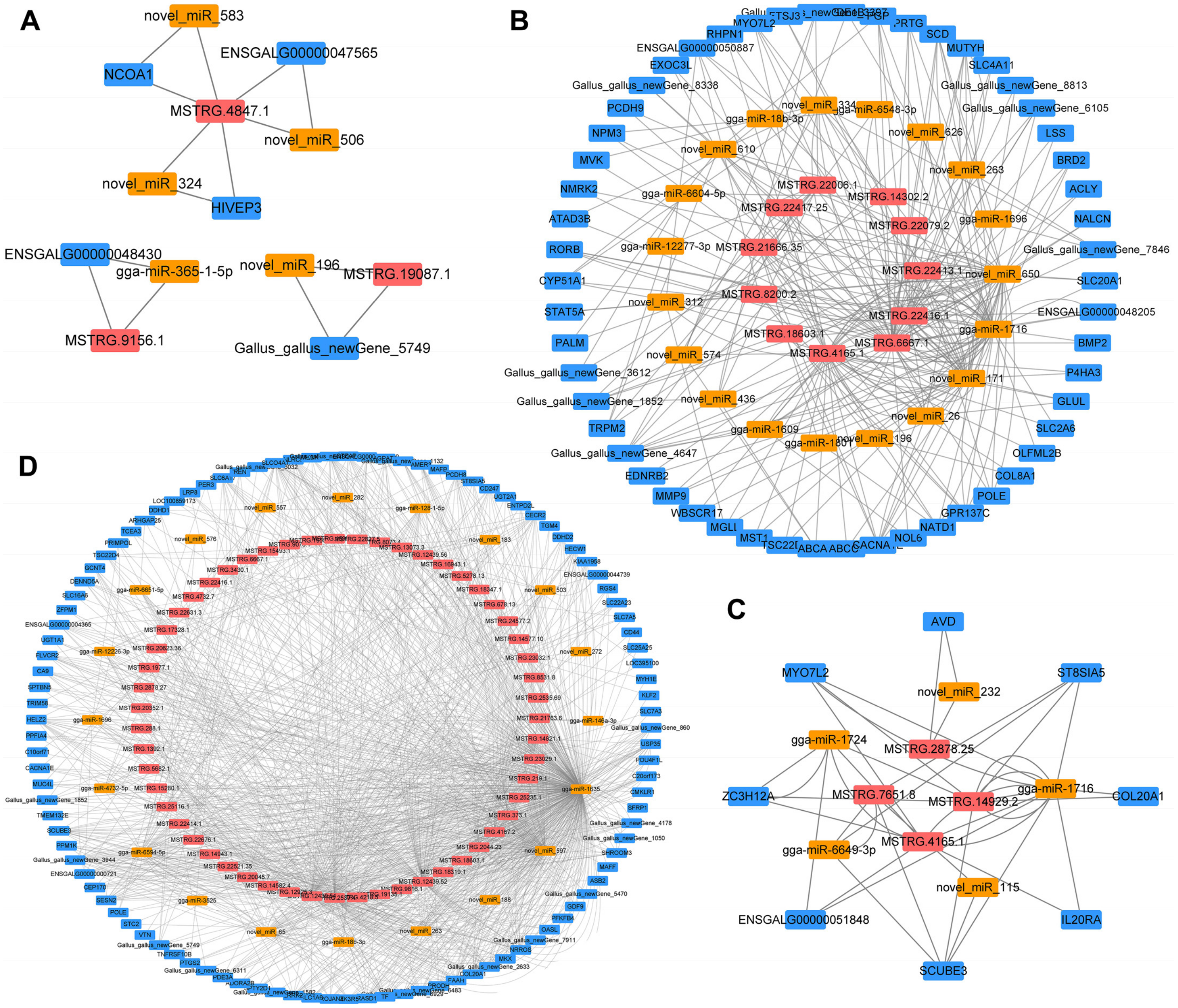

Figure S5. The lncRNA-miRNA-mRNA ceRNA networks in chicken abdominal adipocytes from different differentiation stages. (**A**) The lncRNA-miRNA-mRNA ceRNA networks in A0 vs. A12. (**B**) The lncRNA-miRNA-mRNA ceRNA networks in A12 vs. A48. (**C**) The lncRNA-miRNA-mRNA ceRNA networks in A48 vs. A72. (**D**) The lncRNA-miRNA-mRNA ceRNA networks in A72 vs. A120.
